# Molecular docking analysis of two bioactive molecules KLUF10 and KLUF13 isolated from the marine bacteria Micrococcus sp. OUS9 with TNF alpha

**DOI:** 10.6026/97320630017530

**Published:** 2021-05-31

**Authors:** Shanthi Kumari, Pabba Shivakrishna, K Sreenivasulu

**Affiliations:** 1 Osmania University, Department of microbiology, Hyderabad, India; 2 Lavin laboratories, Hyderabad, India; 3 KLEF University, Andhra Pradesh, India

**Keywords:** TNF-α, KLUF10, KLUF13, PDB ID 2AZ5, Docking

## Abstract

Tumor necrosis factor-alpha (TNF-α) is known to be linked with tumor. Therefore, it is of interest to document the Molecular docking analysis of two bioactive molecules KLUF10 and KLUF13 isolated from the marine bacteria Micrococcus sp. OUS9 with TNF alpha. We report the molecular interactions of KLUF10 and KLUF13 with TNF alpha.

## Background:

Oceans occupy about 70% of the Earth's surface area, and many aquatic species have novel components that are not found on land, as well as specific biological properties of high activity and efficacy. Since the National Cancer Institute (NCI) began screening marine tools for anti-cancer activity in 1968, research on marine drugs has evolved into a distinct field [1]. Docking is a process that predicts the preferred orientation of one molecule to another when they are bound together to form a stable complex [[2] and it plays an important role in drug rational design. The in-silico method is a low-cost and fast approach for identifying protein targets of natural based ingredients [3] By analyzing the interactions between small molecule ligands and receptor biomacromolecules, this method could predict the binding mode and affinity strength, and then realize structure-based drug design, which is of great significance to the molecular mechanisms of pharmacological activities, structure prediction of protein-ligand complexes, and targeted drug screening [4,5,6] A number of molecular modeling and docking studies have been done for predicting molecular targets and molecular mechanism of ginsenosides [7,8,9,10] The most widely used computational technique for characterization of protein-ligand binding sites is molecular docking. A variety of molecular simulation and docking experiments have been conducted in order to predict ginsenoside molecular targets and molecular mechanisms [7,8]. Therefore, it is of interest to document the Molecular docking analysis of two bioactive molecules KLUF10 and KLUF13 isolated from the marine bacteria Micrococcus sp. OUS9 with TNF alpha.

## Materials and methods:

### Sampling site and sample collection:

Seawater and soil samples were collected from coastal locations of Nellore, Visakha Patnam and Bapatla, under aseptic conditions and were processed within 1-2 hours after collection [11 - check with author][12]. The isolated bacteria were screened and identified by molecular characterization (using 16s rRNA sequencing), as per our previous study [13].

### Extraction of crude extracts:

The shake-flask fermentation was performed using 250ml capacity Erlenmeyer flasks for the selected active bacterial strains, containing 100ml of Zobell broth medium. The pure selected bacteria strain was inoculated with 1ml culture suspension for the sterilized fermented broth. Fermentation was conducted on a rotary shaking incubator by incubating inoculated flasks at 28°C, 250 rpm for five days. The fermentation broth was centrifuged for crude extract preparation at 10,000rpm for 20 min after incubation.

### Purification of the active compounds:

The column chromatography had chosen with silica gel of L00-200µm particle size. The gel was suspended for the packing of the column with petroleum ether. The column was formed by a corning glass tube 40 cm long with a glass stopper at the bottom and an internal diameter of 2.5 cm. The column's final size was 25x2.5 cm. The column had methanol balanced. The sample was not exceeding 5 ml, and the flow rate was kept to 0,2 ml / min, with the chloroform gradient water system methanol (9:1, 7:3, 1:1). Then the column was washed with hexaneand methanol. 10ml fractions were gathered and all the different fractions were analyzed with each solvent scheme. All pooled fractions have been tested with antimicrobial agents. The active fraction was analyzed and characterized by NMR spectroscopy and Mass spectroscopy.

### Docking study

#### Protein preparation

TNF-α (PDB ID 2AZ5) protein X-ray crystallographic structure (resolution 3.0) was obtained from the Protein Data Bank. Water molecules, and other heteroatoms ligands, with chains B, C, and D, were excluded from the protein molecule. The CHARMm force field was used to bind hydrogen atoms to the protein. Using the Accelyrs Discovery studio (version 2.1), energy was minimized using the conjugate gradient procedure with an RMS gradient of 0.01kcal/mol.

#### Ligand preparation:

**Figure 1:**
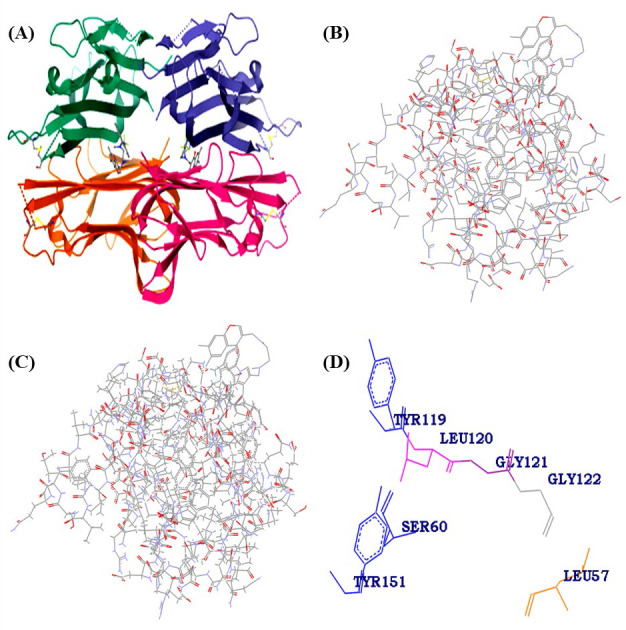
Molecular Docking simulation results: (A) Secondary structure of TNF-α with a small molecule inhibitor (2AZ5), (B) threedimensional structure of 2AZ5_A chain represented in Wireframe model (Black -Carbon,Red-Oxygen,Blue -nitrogen, Yellow-Sulphur), (C) Prepared protein, (D) Shows identification of active site pocket

The ligand molecules (KLUF10-zeaxanthin and KLUF13- novel compound) structure were drawn in Hyperchem molecular modeling and visualization tool (version 7.5) and the energy was minimized using Accelyrs Discovery studio client (version 2.1) software. The minimized protein and ligands were saved in PDB and mol-2 format, respectively for further analysis as shown in the Figure 1 and the energy values obtained.

#### Docking:

The protein and ligand docking studies were performed by using (using a grid-based MD docking algorithm, CDOCKER (CHARMm-based MD docking tool) software (Wu et al., 2003).The CDOCKER interaction energy as an estimation of molecular complex binding affinity was used in this study.

## Results and Discussion:

Marine organisms have developed biochemical and physiological processes including the production of bioactive metabolites for reasons such as reproduction, communication, and predation, infection, and competition protection (Halvorson, 1998). Virtually, every kind of marine organism displays different molecules with distinctive structural characteristics, due to their physical and chemical circumstances in the marine environment. In the present investigation, the total 29 different bacterial cultures were isolated from different locations of Nellore district regions the bacterial colonies present on agar plates with morphologically different pigment producing has been identified. The chosen colonies were screened by well diffusion for antagonistic activity and identified by 16s molecular identification method. The crude extract form Micrococcus sp. OUS9 was separated using column chromatography and from the different fractions obtained from the column the KLUF10 and KLUF13 were shown bioactive nature compare to other fractions. Hence, fraction KLUF10 and KLUF13 was subjected to further analysis to identify the bioactive compound NMR studies. The two fractions were revealed as KLUF10 (Zeaxanthin) KLUF13(1-(1-(4-methoxyphenyl)-2-(methylamino) ethyl)cyclohexanol).

## Docking studies:

**Figure 2:**
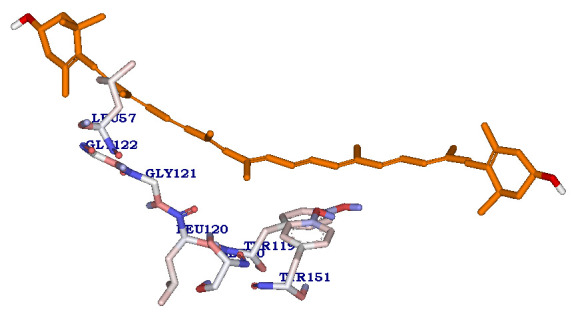
Molecular Docking results of compound KLUF10, showing Receptor-ligand interactions of KLUF10 (Zeaxanthin) compound with active site residues of protein

**Figure 3:**
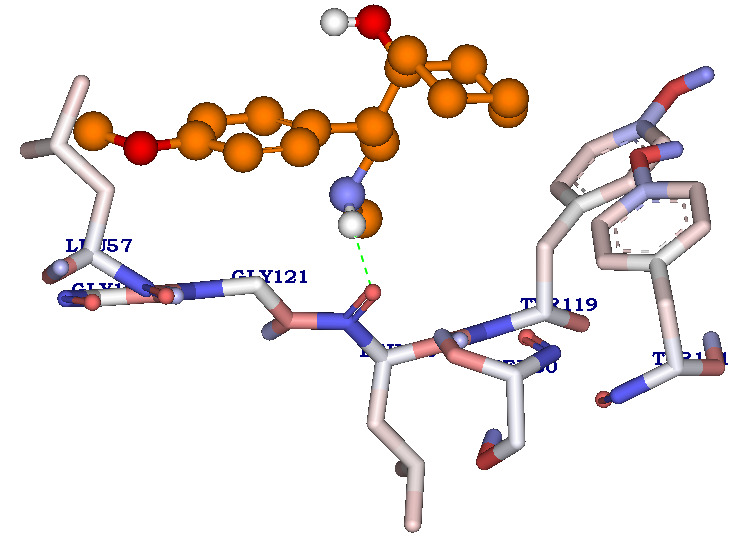
Molecular Docking results of compound KLUF13. Receptor-ligand Hydrogen bonding (green dotted line) interactions betweenKLUF13 (1-(1-(4-methoxyphenyl)-2-(methylamino) ethyl) cyclohexanol) compound with active site residues of protein

Two bioactive compounds isolated from marine bacteria Micrococcus sp. OUS9 KLUF10 and KLUF13substances were selected as possible lead structures and optimized. The inhibitory potential of the obtained structures to PDB ID 2AZ5 were evaluated by means of molecular dockingusing a grid-based MD docking algorithm, CDOCKER (CHARMm-based DOCKER), which offers all the advantages of full ligand flexibility (including bonds, angles, dihedrals). From the docking studies KLUF10 shows the negative CDOCKER energy score (-95.449 K.cal/mol) with binding energy of -41.21210 K.cal/mol (Table 1 - see PDF), and forms non-bonded interactions with active site residues Tyr119, Leu120, Gly121, Gly122, Ser60, Leu57, Tyr151 (Figure 2). Whereas another compound KLUF13 shows CDOCKER energy score (7.235K.cal/mol) with binding energy of -36.84683K.cal/mol with one Hydrogen bonding interaction (Figure 3). Hydrogen bond is formed between the oxygen atom of LEU120 interacting with NH atom of the KLUF10 (C4:H30 - A: LEU120: O) with a hydrogen bond distance of 2.022Å.

## Conclusion:

We report the optimal molecular interactions of KLUF10 and KLUF13 with TNF alpha for further considerations.
